# Clinical predictors and concomitant antiseizure medications effects on seizure control in relation to plasma cenobamate concentration: a multicenter retrospective study

**DOI:** 10.3389/fphar.2025.1668382

**Published:** 2025-09-22

**Authors:** Francesca Felicia Operto, Viviana Izzo, Giovanni Assenza, Anna Chiara Balsamo, Laura Canafoglia, Emanuele Cerulli Irelli, Albino Coglianese, Carlo Di Bonaventura, Antonio Gambardella, Claudio Liguori, Grazia Maria Giovanna Pastorino, Marianna Pezzella, Rosaria Renna, Ilaria Sammarra, Mario Tombini, Mariana Fernandes, Matteo Antonucci, Bruno Charlier

**Affiliations:** ^1^ Department of Health Sciences, “Magna Graecia” University, Catanzaro, Italy; ^2^ Department of Medicine, Surgery and Dentistry “Scuola Medica Salernitana”, University of Salerno, Salerno, Italy; ^3^ Operative Unit of Clinical Pharmacology, University Hospital “San Giovanni di Dio e Ruggi d’Aragona“, Salerno, Italy; ^4^ Research Unit of Neurology, Department of Medicine and Surgery, Università Campus Bio-Medico di Roma, Rome, Italy; ^5^ Operative Research Unit of Neurology, Fondazione Policlinico Universitario Campus Bio-Medico, Rome, Italy; ^6^ University Hospital “San Giovanni di Dio e Ruggi d'Aragona”, Salerno, Italy; ^7^ Department of Diagnostics and Technology, Fondazione IRCCS Istituto Neurologico Carlo Besta, Milan, Italy; ^8^ Department of Human Neurosciences, Sapienza University, Rome, Italy; ^9^ Postgraduate School of Specialization in Clinical Pathology and Clinical Biochemistry, University of Salerno, Salerno, Italy; ^10^ Institute of Neurology, Department of Medical and Surgical Sciences, “Magna Græcia“ University, Catanzaro, Italy; ^11^ Department of Systems Medicine, University of Rome Tor Vergata, Rome, Italy; ^12^ Neurology Unit, University Hospital of Rome Tor Vergata, Rome, Italy; ^13^ Antonio Cardarelli Hospital, Naples, Italy; ^14^ Neurological Clinic and Stroke Unit, AORN San Pio, Benevento, Italy

**Keywords:** drug-resistant epilepsy, cenobamate, plasma concentration, drug-drug interaction, phenotype-drug interaction, seizure management

## Abstract

**Introduction:**

Cenobamate (CNB) is a novel antiseizure medication (ASM) approved as an add-on therapy for drug-resistant focal onset seizures. Although its mechanism of action is not fully understood, CNB enhances inhibitory GABAergic transmission and blocks voltage-gated sodium channels. Its non-linear pharmacokinetics and strong modulation of cytochrome P450 enzymes may significantly affect the metabolism of co-administered ASMs, posing important challenges for polytherapy management. This retrospective study, complemented by prospective follow-up evaluations, aimed at elucidating, in a cohort of subjects enrolled in an Italian multicenter study, the following: i) the CNB dose associated with clinical response; ii) inter-individual variability in CNB plasma concentrations (CNBp); iii) the potential correlation between CNBp and clinical features including age, sex and body mass index (BMI); iv) interactions between CNB and co-administered ASMs.

**Methods:**

We enrolled 53 adults with drug-resistant focal epilepsy, who started CNB with titration to 200 mg/day, were monitored monthly, and classified as responders (≥50% seizure reduction) or non-responders (<50%). In responders, plasma CNB concentration (CNBp) was measured at the time of their first documented clinical response. Multiple linear regression, including covariates such as age, sex, BMI, CNB dose, and co-administered ASMs, was applied to assess independent predictors of CNBp.

**Results:**

A clinical response was achieved in 26/53 (49%) subjects. Among responders, 53.8% (14/26) achieved response on ≤100 mg/day, with CNBp of 0.5–17.6 μg/mL (median ∼5–6 μg/mL). There was no significant relationship between age and gender, although there was a statistically significant correlation between CNBp and BMI (p = 0.038; R2 = 0.157). In subjects co-administered benzodiazepines, zonisamide, phenobarbital, and perampanel, higher CNBp were observed; conversely, lower CNBp were linked to brivaracetam, topiramate, lamotrigine, and levetiracetam. Additionally, a modest decrease in CNBp was associated with carbamazepine, consistent with its known enzyme-inducing effect.

**Discussion:**

These findings suggest that CNB may achieve clinically meaningful seizure control at relatively low doses, with variability in plasma concentrations largely influenced by concomitant medications and, to a lesser extent, by individual characteristics. Despite the limited sample size, our results highlight the value of therapeutic drug monitoring and individualized titration to optimize CNB therapy in drug-resistant focal epilepsy.

## 1 Introduction

Epilepsy, a chronic and disabling disease, is the third most common neurological disorder worldwide. It has a detrimental impact on quality of life and leads to a significant loss of healthy life years, with more than 13 million disability-adjusted life-years (DALYs). Epilepsy is also associated with considerable mortality, with 126,055 epilepsy-related premature deaths reported in 2016 (Beghi et al., 2019). Despite the availability of many anti-seizure medications (ASMs), about 30%–40% of people with epilepsy, especially those with focal onset seizures (FOS), fail to achieve seizure freedom and experience uncontrolled seizures due to drug-resistance (Perucca et al., 2020).

Cenobamate (CNB) is a novel ASM that was approved in 2019 by the U.S. Food and Drug Administration (FDA) to treat uncontrolled FOS in adult individuals. Since 2021, CNB has been approved in Europe as an adjunctive therapy for FOS with or without progression to bilateral tonic-clonic seizures in adult people with drug-resistant epilepsy who have not been adequately controlled despite a history of treatment with at least two appropriately chosen and well-tolerated ASMs (Kwan et al., 2010; Bialer et al., 2013; Roberti et al., 2021).

CNB’s mechanism of action is not yet fully understood, but it seems to have a multi-modal and synergistic effect on two different targets, such as inhibition of voltage-gated sodium channels and positive allosteric modulation at presynaptic and extrasynaptic GABA-A receptors, thus reducing excitatory neurotransmission (Guignet et al., 2020; Barbieri et al., 2023). The pharmacokinetics of CNB are non-linear in distribution and elimination, so the drug half-life increases following dosage, from 30 h after a dose of 10 mg–76 h after 750 mg (Zaccara et al., 2021). According to the prescribing recommendation, CNB is included as an add-on drug; therefore, it is important to know the pharmacokinetic interactions with other ASMs and their eventual dose adjustments. In fact, due to its hepatic metabolism, CNB can alter the simultaneous exposure to other drugs, such as lamotrigine (LTG), carbamazepine (CBZ), phenytoin (PHT), phenobarbital (PB) and clobazam (CLB). Similarly, these ASMs can alter the patient’s exposure to CNB (Roberti et al., 2021).

Although safety and tolerability have been demonstrated in several clinical trials (Elliott et al., 2022; Rodríguez-Uranga et al., 2024; Saouda et al., 2025), CNB treatment still carries a risk of adverse effects. Therefore, the evaluation of CNB plasma concentration in patients is essential to determine the optimal therapeutic regimen for each individual (Steinhoff et al., 2024) and to provide useful information on its bioavailability under different conditions (Charlier et al., 2022). Such benefits can be obtained by fine-tuning and adjusting the dosage of CNB and concomitant ASMs. Given the complex pharmacokinetics, such as the long half-life and interaction with other ASMs, the optimization of CNB’s therapeutic dosage is an intriguing area of investigation. The balance between maximizing clinical benefits and minimizing side effects requires cautious therapeutic management. The challenge of handling add-on therapy stems from CNB’s ability to function as both an inducer and an inhibitor of cytochrome enzymes, specifically CYP2C19 and CYP3A4. The main metabolic processes for CNB are oxidation and glucuronidation. It is a substrate of CYP enzymes, such as CYP2E1, 2B6, 2C19, and 3A4, as well as UGT2B7. Crucially, CNB inhibits CYP2C19 and strongly induces CYP2C8, 3A4, and 2B6, which may have an impact on the exposure of co-administered medications. Clinically significant interactions between drugs could result from this intricate enzyme regulation, especially in polytherapy (Knox et al., 2024).

Most studies suggest that concomitant ASMs do not significantly affect CNB plasma concentrations. However, isolated reports have described potential interactions with specific drugs such as clobazam (CLB), phenobarbital (PB), brivaracetam (BRV), carbamazepine (CBZ), clonazepam, perampanel (PER), and lacosamide (LCM) (Roberti et al., 2021). Therefore, drug–drug interactions should be carefully evaluated in cases of polytherapy, particularly at CNB dosages greater than 300 mg. Current literature lacks evidence for managing co-administered ASMs during CNB titration. Furthermore, the possible impact of inter-individual variability and the patients’ physical characteristics should be considered.

In this context, our study aimed to elucidate the following: i) the CNB dose associated with seizure freedom; ii) inter-individual variability in CNB plasma concentrations (CNBp); iii) the potential correlation between CNBp and clinical features including age, sex, and body mass index (BMI); and iv) interactions between CNB and co-administered ASMs.

## 2 Materials and methods

### 2.1 Study design and participants enrollment

From May 2024 to November 2024, data were collected retrospectively from medical records and complemented by prospective follow-up evaluations. Eligible participants were individuals aged 18 years or older diagnosed with drug-resistant focal epilepsy, who were all receiving a stable regimen of ASMs before the beginning of the study. Patients were enrolled at the Operational Unit of Pediatric Neuropsychiatry, Department of Health Sciences, University of Catanzaro “Magna Graecia”, a specialized epilepsy center that also provides continuity of care for young adults.

The data here described are part of the study entitled “A multicenter, retrospective study to evaluate the pharmacological exposure to cenobamate in patients with epilepsy”, which was conducted in accordance with the guidelines of the International Conference on Harmonization of Good Clinical Practices, as well as the ethical criteria of the Helsinki Protocol (Dixon, 1999; World Medical Association, 2013). The study protocol was approved by the institution’s ethical committee. Written informed consent was obtained from each study participant, as detailed in Section 2.4.

The diagnosis of drug-resistant epilepsy was established according to the criterion of persistent uncontrolled seizures despite treatment with at least two well-tolerated and appropriately chosen ASMs over the past 2 years. Each enrolled individual started treatment with CNB at the recommended initial dose of 25 mg/day, with progressive dose increments in accordance with the standardized titration protocol (T0), until the target maintenance dose of 200 mg/day was reached. The titration phase generally lasted 20 weeks, followed by a 20-weeks maintenance phase, with minor modifications according to individual response and physicians’ discretion (Kwan et al., 2010).

Patients or their caregivers were surveyed monthly to report seizure frequency. Seizure-freedom was defined as the complete absence of seizures between two follow-up visits, which were usually each 28 days. All patients in our cohort were classified according to their response to CNB treatment. Individuals who experienced a ≥50% reduction in seizure frequency compared to baseline were defined as responders (Res), in accordance with widely accepted criteria in epilepsy clinical trials and real-world studies (Roberti et al., 2024). Subjects with a reduction <50% were classified as non-responders (No-res).

The 2-month time point was selected to evaluate the occurrence of early ADRs, since all recruited subjects would have achieved the maintenance dose if they followed the standard titration plans. Although a fixed dosage regimen was initially planned for every patient, official guidelines allowed physicians to adjust titration protocols at their discretion if tolerance problems arose or if a notable clinical response was obtained prior to the scheduled follow-up. This accounts for the observed variations in dose and treatment regimens.

Check points for subsequent assessments were established according to the research center’s outpatient schedule. CNBp were measured once individuals achieved a ≥50% reduction in seizure frequency, while no assessment was performed in No-res group. BMI was monitored throughout the titration and maintenance phases, and further dose adjustments were decided based on individual efficacy and tolerability once the target maintenance dose of 200 mg/day was reached. As an exploratory analysis, potential exposure–response correlations were evaluated only in Res group, using the plasma concentration acquired at the time of their first documented therapeutic response to CNB. This strategy was intended to provide descriptive information on exposure at the point when clinical benefit could reasonably be attributed to CNB, although the exploratory nature of this approach limits its generalizability.

### 2.2 Sample collection and analysis

Whole-blood samples were collected in BD Vacutainer^®^ tubes containing 2,2′,2″,2‴-(Ethane-1,2-diyldinitrilo) tetra acetic acid (EDTA) as an anticoagulant. Whole blood was centrifuged at 3,500 × g for 6 min; resulting plasma was transferred into clean safe-lock tubes and stored at −20 °C until use. CNB and other ASMs medications plasmatic concentration were evaluated through a UHPLC-MS/MS method according to Charlier and coworkers (Charlier et al., 2022) and to Molteni and coworkers (Molteni et al., 2024). LC-MS/MS analysis was carried out on a Waters Xevo TQ-XS triple quadrupole mass spectrometer coupled to a Waters Acquity UPLC I-Class System (Waters Corporation, Sesto San Giovanni, Italy).

Ultra-pure solvents and formic acid were purchased from Romil (Waterbeach Cambridge, GB); CNB (100 mg) was purchased from DC Chemicals^®^ (Shanghai, China). The deuterated IS lamotrigine-13C3-d3 (1 mg) was obtained from LGC standards (LGC^®^ Group).

### 2.3 Data collection and statistical analysis

Data from electronic medical records were culled for baseline and subsequent evaluation. If more than one follow-up occurred within one of the defined study intervals, the mean seizure frequency was calculated. We used current International League Against Epilepsy (ILAE) guidelines to classify seizure types and epilepsy (Scheffer et al., 2017; Wirrell et al., 2022).

At enrollment, data on demographic characteristics (age, sex, and BMI), seizure etiology, previous and concomitant ASMs, and previous history of epilepsy surgery or neuromodulation were collected. At each follow-up, patients were interviewed and examined for seizure frequency, CNB dose modifications, concomitant ASM doses, BMI changes, and possible CNB discontinuation. Additionally, in patients who achieved clinical response, the CNB dosage and corresponding CNBp at the time of the first observed response were collected.

The associations between the variables and clinical response were tested using Chi-square tests and Student’s t-tests. Pearson’s and Spearman’s correlation were used to quantify the strength and direction of associations, and the response prediction study was conducted using linear regression tests. Multiple linear regression models were used in multivariate analysis to assess the independent effects of pharmacological and clinical variables on CNBp. Age, sex, BMI, CNB dosage, and the presence of enzyme-inducing ASMs (e.g., CBZ, PB, PHT) were among the covariates involved in the model. The assumptions of the model were examined for homoscedasticity, multicollinearity (VIF < 2), and normality. P-values less than 0.05 were considered statistically significant. Cohen’s d was calculated to estimate the effect size for each co-administered drug on CNBp. All analyses were performed using R Statistical Software (v4.1.2; R Core Team 2021).

### 2.4 Ethical implications

This study is part of an ambispective observational protocol and was approved by the local ethics committee (*Registry Protocol No. 101 of 28 March 2024 of the TERRITORIAL ETHICS COMMITTEE OF THE CALABRIA REGION. Established with DDR No. 7927 OF 07/06/2023 Department of Health and Welfare1*). Patients’ informed consent was acquired at the time of the enrollment.

## 3 Results

### 3.1 Clinical features

Table 1 summarizes the demographic and clinical data of our study population. As detailed in the Materials and Methods section, 26/53 (49.1%) enrolled individuals were classified as Res and 27/53 (50.9%) as No-res. No statistically significant differences were observed between the two groups (Table 2).

**TABLE 1:** Demographical and clinical features of enrolled individuals.

	Drug-resistant focal epilepsy n° = 53
Sex, n° (female %)	28 (52.8%)
Age, years	41 ± 17
Age at onset, years	15 ± 15
Epilepsy duration, years	31 ± 17
Etiology, n° (%)	
Structural	29/53 (54.7%)
Genetic	9/53 (17%)
Unknown	15/53 (28.3%)
BMI	26 ± 7

**TABLE 2:** Clinical characteristics stratified by treatment response.

	Responders n° 26	Non-responders n° 27	p-value
Sex, n° (female %)	15 (57.7%)	13 (48.1%)	0.50
Age, years	45 ± 18	36 ± 16	0.06
Age at onset, years	14 ± 15	15 ± 15	0.97
Epilepsy duration, years	33 ± 18	27 ± 15	0.03
BMI	27 ± 5	25 ± 8	0.40
ASMs, n° (at T_0_)	3 ± 1	3 ± 1	0.68

### 3.2 Treatment response and CNB exposure

**FIGURE 1:**
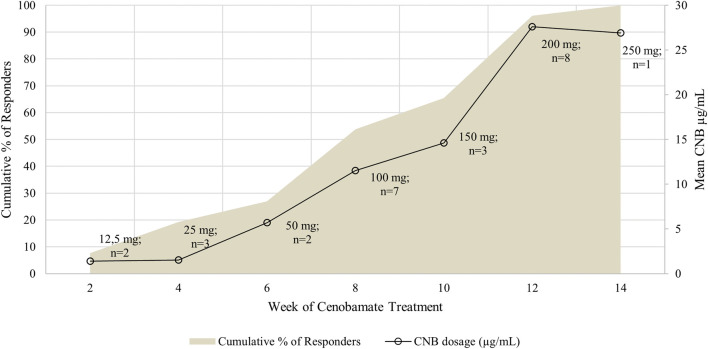
Time to response plot showing the weekly cumulative responder rate and CNB doses at response. The shaded area represents the cumulative percentage of patients achieving ≥50% reduction in seizure frequency over time. Data points indicate the week of treatment, the corresponding CNB dose, and the number of patients achieving response at each level.

Data were collected from medical records over a 6-month period. Fifty-three individuals were included in the analysis, and 26 (49.1%) responded to CNB add-on treatment. At the first documented clinical response (>50% seizure reduction), the mean CNB dose was 122.1 ± 83.1 mg/day (range 12.5–300), while the mean CNBp was 14.1 ± 11 μg/mL (range 0.5–29.6). Figure 1 illustrates the time to response and corresponding CNBp values.

We subsequently analyzed the14 of 26 responders (53.8%) who achieved seizure control with ≤100 mg/day of CNB. Most of them (71.4%) were on polytherapy with three or more ASMs, while the remaining 28.6% were taking two additional ASMs. The most frequently co-administered drugs were carbamazepine (CBZ) and brivaracetam (BRV), each prescribed in six individuals, often in association with valproate (VPA), clobazam (CLB), or eslicarbazepine (ESL). CNBp values in this subgroup ranged from 0.5 to 17.6 μg/mL (median ≈ 5–6 μg/mL), showing considerable interindividual variability. Notably, some patients treated with ≤100 mg/day reached plasma concentrations comparable to those usually observed at ≥200 mg/day; these individuals were commonly co-treated with combinations including sodium channel blockers (e.g., CBZ or ESL) together with VPA, BRV, or CLB. Further details on concomitant ASMs in low-dose responders are provided in Supplementary Figure S1.

### 3.3 Correlation between CNBp and clinical variables

Several clinical variables, including weight, age, sex, and BMI, were analyzed to assess their potential influence on CNBp. Correlation analyses were performed using both dose-normalized and non-normalized CNBp values to explore possible associations between plasma concentration and these individual-related variables. When using dose-normalized CNBp, no significant correlations were found with age (p-value = 0.837), BMI (p-value = 0.985), and sex (p-value = 0.978). When non-normalized CNBp data were used, BMI showed a statistically significant correlation (p-value = 0.038, R2 = +0.157), while age (p-value = 0.317) and sex (p-value = 0.126) remained non-significant. The possible impacts of age, body mass index (BMI), and sex on CNBp concentrations were evaluated using multiple linear regression. A binary variable (0 = male, 1 = female) was used to code sex (Table 3).

**TABLE 3:** Multiple linear regression analysis of the effects of sex, BMI, and age on CNBp.

Variable	Effect size	p-value	95% CI lower	95% CI upper
Intercept	2.392	0.8602	−25.930	30.715
Sex	−1.793	0.7385	−12.982	9.395
BMI	0.938	0.0743	−0.104	1.979
Age	−0.312	0.1139	−0.709	0.084

### 3.4 Influence of concomitant ASMs on CNB response

After classifying patients as Res and No-res, we investigated the potential influence of co-administered ASMs on clinical outcomes. Our data highlighted that VPA, levetiracetam (LEV) and CBZ were the most commonly used medications in both groups, although dosages varied considerably among patients and across follow-ups, which posed a significant challenge for statistical analysis. VPA was the most frequently used drug in both groups, with a 46% higher prevalence in the No-res group compared to Res. The mean VPA doses in the No-Res and Res groups were 1,100 mg and 1,277 mg, respectively. Other commonly used medications included PB, CBZ, and LCM. As expected, the No-res group tended to increase the VPA dosage during follow-ups. Our data showed that there was no clear relationship between the number of administered ASMs and seizure reduction. At treatment initiation and at the time of the first documented clinical response (1R), mean changes in ASM doses were recorded. The following variations (in milligrams) were observed between T0 and 1R.

• LCM (Lacosamide): +106.2 mg
• LEV (Levetiracetam): +333.33 mg
• BRV (Brivaracetam): 0 mg (no change)
• OXC (Oxcarbazepine): −360 mg
• ESL (Eslicarbazepine): −533.33 mg
• CBZ (Carbamazepine): −285.71 mg
• LTG (Lamotrigine): 0 mg (no change)
• RUF (Rufinamide): +1,200 mg
• PER (Perampanel): −1.67 mg
• GBP (Gabapentin): 0 mg (no change)
• PB (Phenobarbital): 0 mg (no change)
• FBM (Felbamate): −2,400 mg
• ZNS (Zonisamide): −100 mg
• VPA (Valproate): −128.57 mg
• TPM (Topiramate): −50 mg

Dose adjustments of concomitant ASMs varied widely, with some drugs showing increases (e.g., RUF, LEV), others reductions (e.g., ESL, FBM), and several remaining unchanged. No clear correlation emerged between dose variations and clinical response. The mean difference in CNB dosage required to achieve a ≥50% seizure reduction in relation to co-administered ASMs is shown in Supplementary Figure S2. However, given the small number of patients, no statistically significant changes were observed.

Exploratory analyses of the effect of co-administered ASMs on CNBp showed heterogeneous results. Large effect sizes (Cohen’s d) were observed for BNZ (d = 1.37), ZNS (d = 0.98), PB (d = 0.94), and PER (d = 0.84). Smaller positive effects were found for OXC, d = 0.34), VPA (d = 0.31), LCM (d = 0.19), and CLB (d = 0.06). Negative associations were seen for CBZ (d = −0.09), ESL (d = −0.21), LEV (d = −0.39), TPM (d = −0.55), LTG (d = −0.57), and BRV (d = −0.82). These findings point to potential variations in CNBp with co-medications, although the small sample size warrants cautious interpretation.

### 3.5 Demographic predictors of clinical response to CNB

**FIGURE 2:**
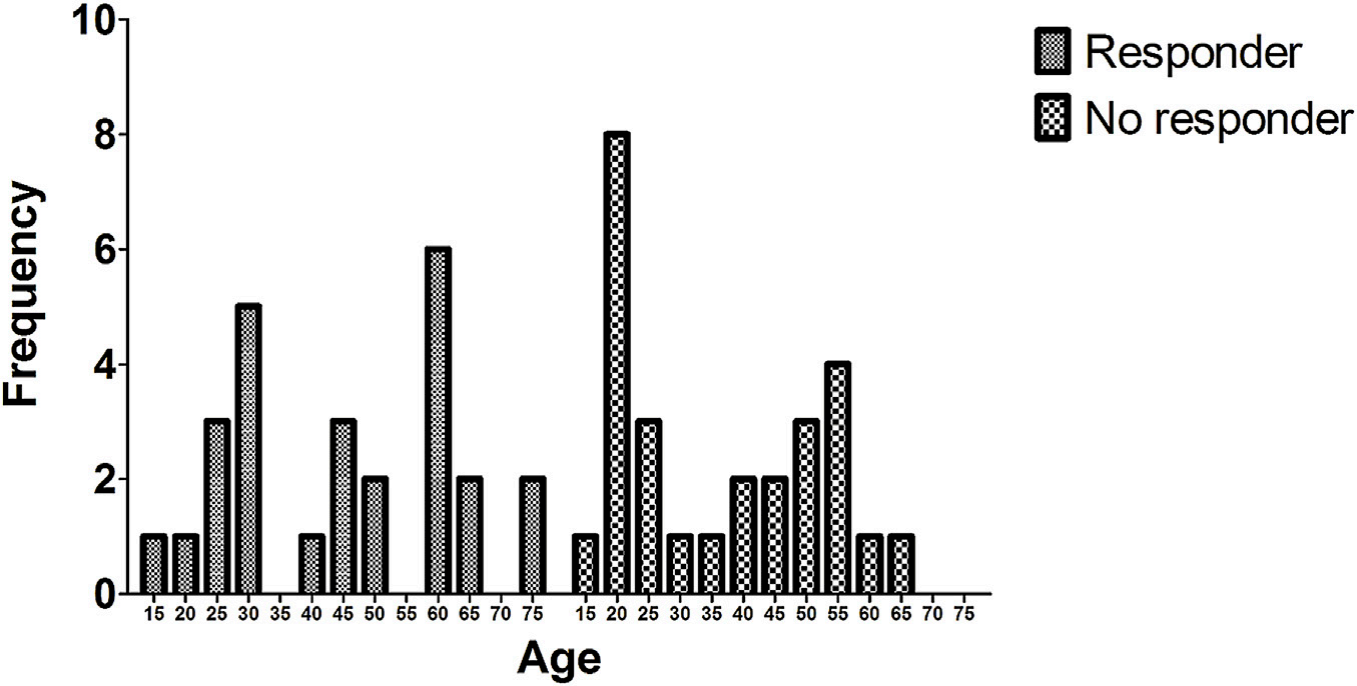
Age distribution in responder and non-responder groups. The histogram displays the frequency of patients across age intervals for responders (striped bars) and non-responders (checkered bars).

We also explored the relationship between age and treatment response. As shown in Figure 2, Res patients were older on average (45.2 ± 17.5 years) than No-res (36.3 ± 16.5 years). This suggests that age may play a role, with older patients tending to respond better. However, the wide variability within groups and the small sample size limits the strength of this conclusion.

**FIGURE 3:**
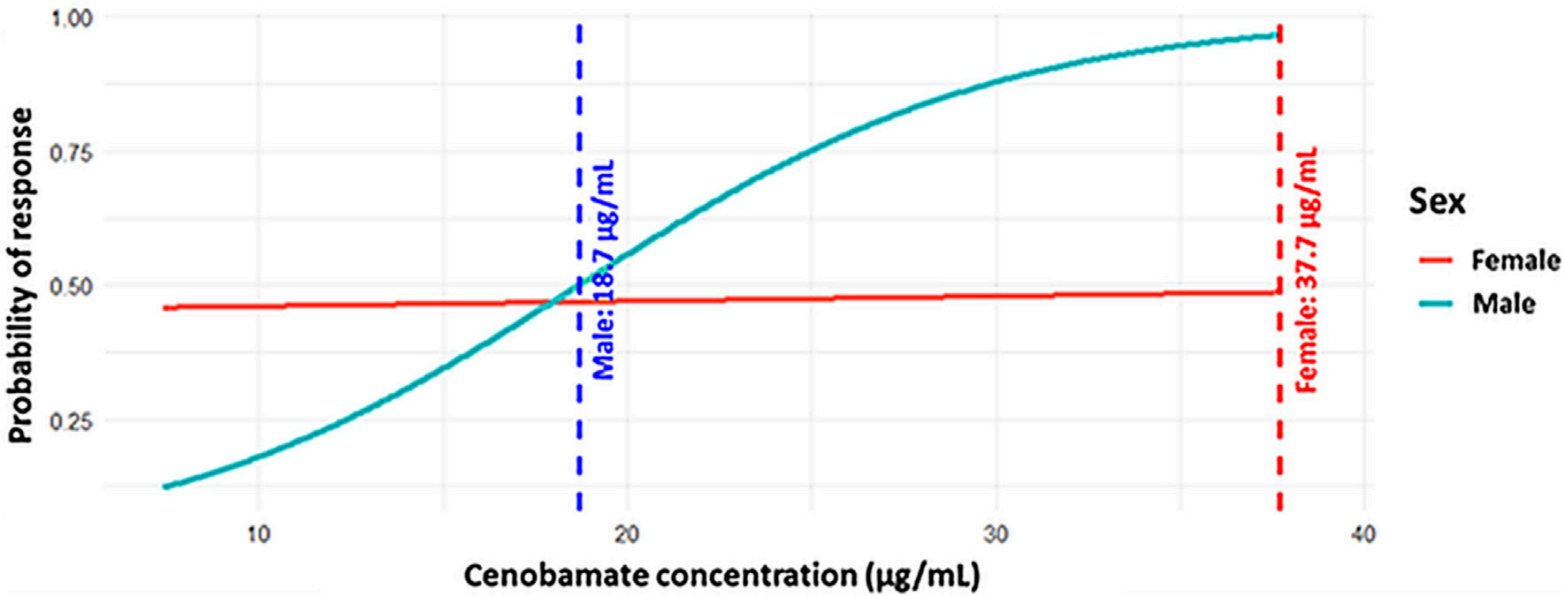
Predicted probability of response by sex. The plot displays the concentration–response curve for males and the flat probability curve for females, with vertical dashed lines indicating mean effective concentrations (18.7 μg/mL for males and 37.7 μg/mL for females).

Gender may have also a significant impact on treatment response. In our sample, males seemed to reach the ≥50% response threshold at lower CNB plasma concentrations compared to females, who required nearly twice the concentration (Figure 3). This difference was not statistically significant (p = 0.126), but it suggests possible pharmacokinetic or pharmacodynamic differences between sexes.

Neither the Chi-square test (p-value = 0.674) nor the Fisher’s exact test (p-value = 0.586) revealed a statistically significant association between sex and responder status overall. This may be explained by the sex-related difference becoming apparent only at specific concentration ranges, rather than across the overall distribution.

## 4 Discussion

Our study showed that CNB can reduce seizure frequency even at low doses, regardless of the type or dosage of co-administered ASMs. This finding is limited by the small number of patients but remains consistent with previously published research. Moreover, this effect appeared to be largely independent of individual-related clinical variables, as no significant correlations were found between plasma CNB concentrations and factors like age, sex, or BMI. When plasma concentrations were evaluated without adjusting for dose, a weak correlation with BMI and body weight emerged, suggesting that anthropometric factors may influence drug exposure. Individuals with a higher BMI showed a slight positive trend toward achieving clinical response. The significant correlation between non-dose-normalized CNBp and BMI suggests anthropometric factors may affect drug exposure. The wide variability observed in CNBp, even at comparable CNB doses, may also be influenced by additional factors such as treatment adherence, concomitant polytherapy, and individual genetic differences in drug metabolism. No significant associations were found for age or sex. Furthermore, our data indicated a potential sex-related trend in therapeutic response, with males achieving a ≥50% seizure reduction at lower CNB plasma concentrations than females. This difference was not statistically significant, but it suggests possible sex-specific pharmacokinetic or pharmacodynamic differences that warrant further investigation in larger samples.

Nonetheless, co-medications appeared to influence CNB plasma concentrations, with potential implications for dose titration and therapeutic drug monitoring. Subjects receiving enzyme-inhibiting co-medications (e.g., ZNS, PB, PER) may reach higher CNBp concentrations at the same dose, allowing for lower titration targets and potentially reducing adverse effects such as somnolence, dizziness, or ataxia (Roberti et al., 2021). Conversely, enzyme-inducing or interacting ASMs (e.g., BRV, TPM, LTG, LEV) were associated with lower CNBp concentrations, potentially requiring closer follow-up and more aggressive titration to achieve therapeutic exposure. These considerations are particularly relevant in highly polytreated patients, where the cumulative risk of drug–drug interactions is high.

In the clinical trial C017, 19% of individuals with uncontrolled focal epilepsy became seizure-free from the first day of CNB treatment, and 23.6% remained free from focal seizures for at least 1 year (Brandt et al., 2022). Our findings are consistent with these results, showing that CNB can induce seizure freedom even at very low doses. A Spanish work confirmed the efficacy of CNB in reducing seizure frequency or achieving seizure freedom, independent of the number of co-administered ASMs. In this study, 44.7% of 170 CNB-treated patients experienced a reduction of concurrent ASMs (Villanueva et al., 2023). Dose reduction or withdrawal was particularly common when sodium channel blockers (e.g., PHT, CBZ, OXC, LTG, LCM, clobazam) were used together with CNB, likely due to overlapping mechanisms of action. Similarly, Rosenfeld and coworkers further confirmed that CNB efficacy was unaffected by the amount of co-administered ASMs. Their patients showed that extra CNB led to clinically significant decreases in seizure frequency, including seizure freedom, regardless of the number of concurrent ASMs (Rosenfeld et al., 2021).

Our observations draw attention to the potential therapeutic effect of CNB at sub-therapeutic dosages, particularly when combined with enzyme inhibitors or sodium channel blockers. Nonetheless, these hypotheses remain exploratory, as no statistically significant interactions were detected, likely due to the small sample size.

Our data did not show a significant correlation between CNB dose and seizure reduction. Only a few patients benefited from increasing other ASMs, while clinical improvement was observed mainly after adding CNB, even at initial doses of 25 mg/day. The effectiveness of CNB at low doses has already been reported (Villani et al., 2022; Steinhoff et al., 2024). Although we did not find statistically significant associations between co-administered drugs and the CNB dose needed for response, some agents showed trends toward higher or lower dose requirements. These trends may reflect pharmacokinetic or pharmacodynamic interactions, but our limited sample size prevents firm conclusions. Larger studies are needed to confirm these findings and clarify the underlying mechanisms.

As shown in Figure 1 and Supplementary Material, most patients achieved clinical benefit within 8–12 weeks, with higher response rates observed at moderate CNB doses (100–200 mg/day). Co-administered ASMs influenced the CNB dose required for response. Notably, patients receiving BRV required significantly lower CNB doses, likely reflecting synergistic or dose-sparing effects. This is consistent with Bender et al. (2025), who reported increased BRV levels during CNB treatment, likely due to CYP2C19 inhibition, underlining the importance of therapeutic drug monitoring (Bender et al., 2025). Conversely, CBZ co-treatment was associated with lower CNB plasma concentrations, reflecting its enzyme-inducing properties that may accelerate CNB metabolism. Interestingly, some responders on low CNB doses (≤100 mg/day) reached plasma levels (≥17 μg/mL) usually observed at higher doses, indicating substantial interindividual variability. These cases often involved polytherapy with sodium channel blockers (e.g., CBZ, ESL) combined with agents such as VPA, BRV, or CLB. Overall, these findings highlight the need for dose optimization, careful consideration of drug–drug interactions, and individualized titration strategies. However, the small sample size limits the generalizability of these results.

Notably, two retrospective studies reported different conclusions regarding the effect of increasing CNB dosage. Peña-Ceballos and coworkers found a dose-dependent improvement in seizure control at 200 mg/day, (Peña-Ceballos et al., 2023), whereas the multicenter study by Villanueva did not observe a significant dose-dependent effect on seizure frequency (Villanueva et al., 2023).

Despite these promising findings, our study has several limitations. First, the small sample size limits the generalizability of our results, reducing the statistical power to detect relevant associations between CNB plasma concentrations and clinical variables. Second, the observational design and heterogeneity in co-administered ASMs may have introduced confounding factors that could not be fully controlled. Thirdly, the limited follow-up period (6 months) may not be sufficient to capture long-term efficacy or late-onset adverse effects. Lastly, although females required higher plasma concentrations than males to achieve a clinical response, this difference was not statistically significant and should be interpreted with caution.

## 5 Conclusion

This retrospective analysis did not show clear benefits from increasing the dosage of co-administered ASMs in patients who responded to CNB. For example, adjustments made by physicians, such as increasing RUF and LEV or reducing ESL and OXC, did not consistently correlate with treatment response. In line with recent literature, our cohort indicates that CNB produced a clinical response regardless of the concentration or type of concomitant ASMs. The lack of a strong correlation between increasing ASMs and seizure reduction in Res group raises questions about the complexity of epilepsy management and emphasizes the importance of tailoring to individual responses rather than simply adding or escalating medications. Since CNBp was measured only in responder group, a selection bias may limit the extrapolation of pharmacokinetic findings to the entire cohort. Analyses by treatment and phenotypical characteristics, like BMI, age, sex, revealed no statistically significant differences, suggesting that other factors, possibly related to drug tolerability or individual variability, may influence response. Despite the several limitations of this study, individuals’ chronological age and age at seizure onset may be relevant when evaluating treatment responses and planning epilepsy management strategies, reinforcing the need for individualized approaches across different age groups.

Future studies should include larger sample sizes, prospective design, CNBp monitoring in all patients (regardless of response), and multivariate models to validate these preliminary observations. Notwithstanding the limitations that have already been extensively discussed, our results are in line with recent research and confirm the complexity of CNB pharmacodynamics, emphasizing the need for further researches to clarify the factors influencing its effectiveness, particularly in combination with other ASMs.

## Data Availability

The raw data supporting the conclusions of this article will be made available by the authors, without undue reservation. The data is available by contacting the corresponding author.
