# Supplementary material for: Clinical predictors and concomitant antiseizure medications effects on seizure control in relation to plasma cenobamate concentration: a multicenter retrospective study
Source: Front Pharmacol. 2025 Sep 22;16:1668382. doi: 10.3389/fphar.2025.1668382 (PMC12497770; doi:10.3389/fphar.2025.1668382)
Supplement: Supplementary file 1 [file DataSheet1.pdf]

Supplementary Material

Supplementary Material

1 Supplementary Figures

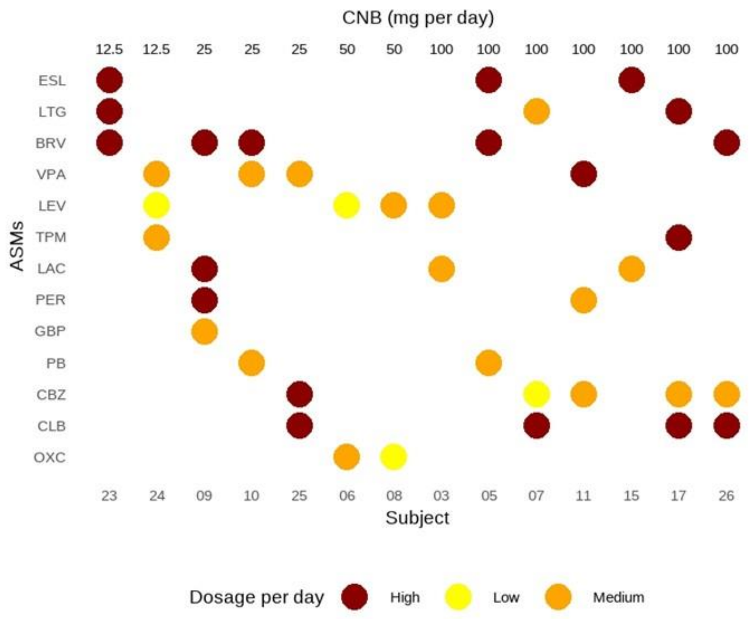

Supplementary Figure 1. Concomitant ASMs in responder individuals at low CNB dosage (≤100 mg/day). Each ASM is represented by a coloured dot, with the colour indicating the prescribed dose category (low, medium, or high) as shown in the legend. Dose categories were defined as follows:
– Low: LEV ≤ 750 mg/day, CBZ: 500 mg/day, OXC: 600 mg/day
– Medium: LEV: 1500 mg/day, PER: 4 mg/day, VPA: 1000-1250 mg/day, LTG: 250 mg/day, PB: 75 mg/day, TPM: 200 mg/day, OXC: 900 mg/die
– High: LCS: 400 mg/day, PER: 8 mg/day, ESL ≥ 1200 mg/day, CBZ ≥ 1200 mg/day, BRV: 200 mg/day, CLB ≥ 20 mg/day, VPA: 2000 mg/day, LTG: 400 mg/day, TPM: 700 mg/day

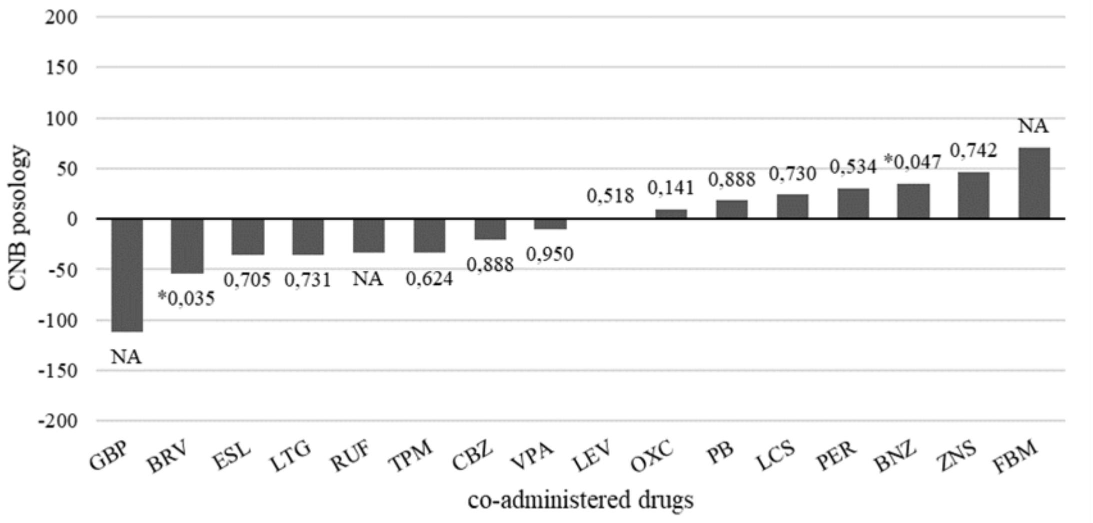

Supplementary Figure 2. Impact of Co-administered Drugs on CNB Dose at Clinical Response. Values above each bar indicate p-values from two-sample t-tests; “NA” indicates insufficient data to compute a valid comparison.

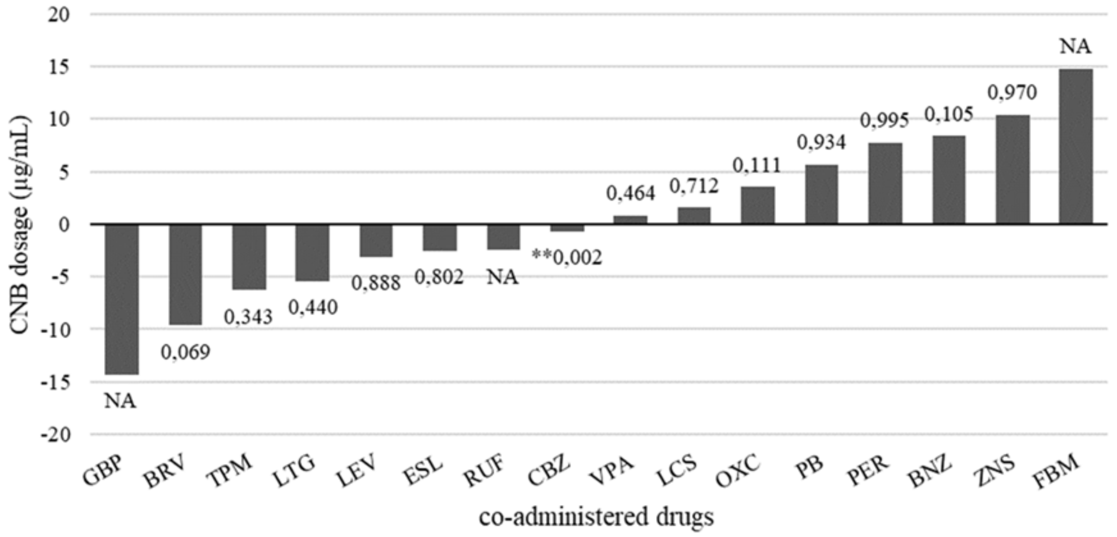

Supplementary Figure 3. Impact of Co-administered Drugs on CNB Plasma Concentration at Clinical Response. Values above each bar indicate p-values from two-sample t-tests; “NA” indicates insufficient data to compute a valid comparison.
